# Effect of pearlitic morphology with varying fineness on the cavitation erosion behavior of eutectoid rail steel

**DOI:** 10.1016/j.ultsonch.2020.105399

**Published:** 2020-11-17

**Authors:** Arun Rajput, J. Ramkumar, K. Mondal

**Affiliations:** a Materials Science Programme, Indian Institute of Technology Kanpur, Kanpur 208016, India; b Department of Mechanical Engineering, Indian Institute of Technology Kanpur, Kanpur 208016, India; c Department of Materials Science and Engineering, Indian Institute of Technology Kanpur, Kanpur 208016, India

**Keywords:** Cavitation erosion, Pearlite, Furnace-cooled, Air-cooled, Hardness, Mean depth of erosion (MDE), Mean depth erosion rate (MDER)

## Abstract

The present work discusses the effect of the pearlitic morphology with varying fineness on the cavitation erosion behavior of eutectoid rail steel. Cavitation erosion of three different types of the pearlitic steels (furnace-cooled, air-cooled, and forced-air-cooled) consisting of coarse, fine, and very fine microstructures were tested in 3.5% NaCl solution and compared with that of the as-received pearlitic rail steel. The variation in the mean depth of erosion (MDE) and mean depth erosion rate (MDER) with erosion time was analyzed. Furthermore, the cavitation erosion resistance of the as-received, the air-cooled, and the forced-air-cooled was found to be 1.03, 1.51, and 2.14 times better than the furnace-cooled pearlitic steel, respectively. It was concluded that the cavitation erosion resistance of the pearlitic steel increased with the increase in the fineness of the microstructure.

## Highlights

• Cavitation resistance of pearlitic steels solely depends on its microstructure.
• Pearlitic steel with finer microstructure shows better cavitation resistance
• The interface of the pearlitic colonies is found to be the first area of attack.
• The cementite phase shows better cavitation resistance than the ferritic phase.
• The nano-indentation test has precisely predicted the cavitation resistance.

## 1 Introduction

Cavitation is defined as a type of erosive wear in which the degradation of the material's surface takes place due to the action of bursting bubbles in an aqueous media. The formation and bursting of fluid bubbles near the material surface is the major source of cavitation erosion wear [1]. Moreover, the change in operating pressure is responsible for the formation and bursting of fluid bubbles [2]. The working pressure fluctuates above and below the saturated vapor pressure of the operating liquid specific to the fluid temperature, and subsequently leads to the change in phases. Moreover, the fluid changes its phase from liquid to vapor when operating pressure drops below its vapor pressure and again converts into the liquid when working pressure rises above its vapor pressure [3]. Furthermore, the powerful waves are generated during the conversion of the vapor phase into the liquid phase due to the sudden reduction in volume [1]. The intensity of these pressure waves is sometimes higher than the strength of the material and leads to catastrophic failure of the material [4], [5]. Repetitive attacks of these pressure waves on the material surface also create deformed anodic and undeformed cathodic zones, and this effect promotes the gradual loss of material due to corrosion. Hence, the material with excellent corrosion resistance is beneficial for use in the cavitation-prone environment [6], [7]. The submerged jets, ship propellers, pumps, and turbines are a few of the fluid operating machineries where the phenomenon of cavitation erosion can be easily spotted [8], [9].

The mechanical properties of the material can be transformed by changing its microstructure with the help of heat treatment [10], [11]. Amongst the mechanical properties, hardness is one such significant property, which effectively predicts the behavior of material against cavitation erosion. Heymann et al. [12] have established the relationship between the hardness and cavitation erosion resistance for nine kinds of metals, which include stainless steel, carbon steels, and other nonferrous alloys, and concluded that the cavitation erosion resistance varies as the 5/2 power of the hardness. S. Hattori et al. [13] have created a database for the carbon steels, and found out that the cavitation erosion resistance of the material is directly proportional to its Vickers hardness. Elasticity is the second crucial mechanical property in determining the behavior of material against cavitation erosion [14]. The high-nitrogen austenitic stainless-steel shows better cavitation erosion resistance after improving its elasticity by solution treatment [14]. The micro galvanic corrosion also takes place at the interface of different phases, and this further exaggerates the loss of material due to the action of corrosion [15]. In the case of cast iron, the ferrite phase is dissolved in the vicinity of graphite nodules because of the formation of a micro-galvanic couple, in which ferrite acts as an anode and graphite nodules act as cathode [15]. A. Al-Hasem et al. [16] have performed cavitation erosion tests on a nodular cast iron. They have reported that the initiation of cavitation damage first occurs at the graphite/ferrite interface because of the micro-galvanic activity.

Pearlitic steels have a high potential as cavitation erosion resistance material because of its right blends of mechanical properties, like strength and toughness [17]. The strength and toughness of the pearlitic steel are directly related to some microstructural parameters, such as prior austenite grain size, pearlitic colony size, and interlamellar spacing [18], [19]. The composition, microstructure, and surface treatment also play vital roles in the performance of a particular material against the cavitation erosion. The near eutectoid steel (0.71% C, 0.24% Si, and 1.14% Mn) as selected in this work is mainly used in manufacturing Indian rail. This steel is usually of pearlitic microstructure [20], and is also used in other structural applications, like springs, high strength wires, and locomotive tires [21]. In this regard, the interlamellar spacings of the pearlite in rebar steel can be modified using different cooling rates after austenitization, and subsequently, its mechanical and corrosion behavior can change significantly [22], [23]. However, the literature does not reveal that any systematic study has been performed on the cavitation erosion behavior of pearlitic steel, such as Indian rail steel, as a function of pearlitic spacings. One of the ways to modify the pearlitic spacings is the cooling rate, and it would definitely be an interesting analysis to understand the effect of cavitation damage as a function of pearlitic spacings or cooling rate.

Therefore, the current paper aims to study the cavitation erosion performance of heat-treated pearlitic Indian Rail steel (0.70 wt% C) as a function of pearlitic spacing. The as-received steel has been exposed to various heat treatments like furnace-cooling, air-cooling, and forced-air-cooling, after austenitization to generate three different pearlitic morphology. Besides, the as-received rail steel is also in pearlitic condition, and the cavitation erosion behavior of the newly developed pearlitic steels has been critically assessed and compared with that of the as-received steel as well. The mechanism of cavitation with interlamellar spacing has also been proposed.

## 2 Experimental procedure

### 2.1 Materials preparation

The steel selected in this work had the following chemical composition: 0.71% C, 0.24% Si, 1.14% Mn, 0.024% P, 0.023% S, 0.013% Cr, 0.014% Ni, 0.011% As, 0.012% Nb, 0.074% Ta and 97.68% Fe (wt.%), with traces of other elements. The samples were cut down in the form of a circular disc from this rail. The dimensions of the sample are as follows: 20 mm in diameter and a thickness of 5 mm. The time-temperature-transformation diagram used to produce the different pearlitic steels was generated with the help of software built by H. Bhadeshia [24]. The lower and upper critical temperature (A_c1_ and A_c3_) for the transformation of austenite into the ferrite and carbide were calculated from Eqs. (1), (2), respectively. The bainite start temperature (B_s_) was calculated from Eq. (3). Besides, the martensite start temperature (M_s_) was calculated from Eq. (4) [25].

(1) A_c1_ (°C) = 723 − 10.7 Mn − 16.9 Ni + 29.1Si + 16.9 Cr + 290 As + 6.38 W

(2) A_c3_ (°C) = 910 − 203 (0.708C)^1/2^ − 15.2 Ni + 44.7Si + 104 V + 31.5 Mo + 13.1 W

(3) B_s_ (°C) = 656 − 57.7C − 35 Mn − 75Si − 15.3 Ni − 34 Cr − 41.2 Mo

(4) M_s_ (°C) = 539 − 423C − 30.4 Mn − 17.7 Ni − 12.1 Cr − 7.5 (Mo, W, Si)

**Fig. 1:**
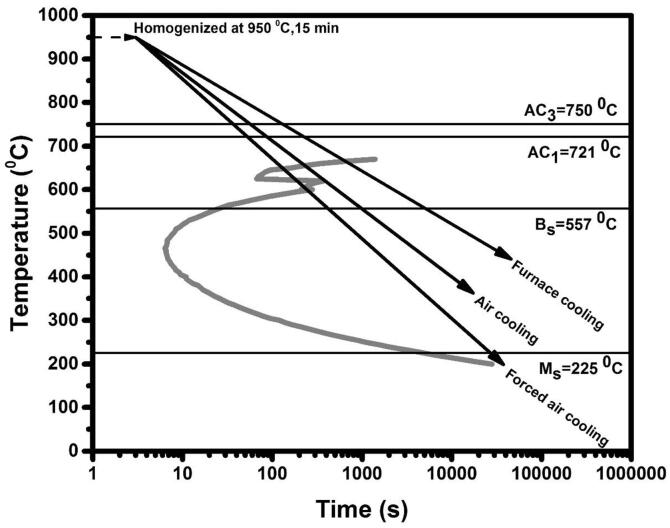
Time-temperature-transformation diagram and subsequent schematic heat treatment procedure for making of different pearlitic steels.

The values of lower (A_c1_) and upper (A_c__3_) critical temperature were found to be 721 and 750 °C, respectively. Whereas, the temperatures of bainite (B_s_) and martensite (M_s_) formation were found out to be 557, and 225 °C, respectively. All the critical temperatures are shown in Fig. 1. The samples were austenitized for 15 min. at 950 °C. After that, coarse pearlite, fine pearlite, and very fine pearlite were produced by furnace-cooling, air-cooling, and forced air-cooling, respectively. The forced air-cooling was carried out by taking the sample after austenitization and keeping it in front of a table fan. The heat treatment processes are shown schematically in Fig. 1.

### 2.2 Mechanical and phase characterization

The Rockwell hardness test was performed to evaluate the micro-hardness of the given pearlitic steels. The samples were polished with the help of emery papers up to 2000 grit size. Subsequently, cloth polishing was also done using 0.05 μm size alumina powder before the hardness test. The C scale Rockwell hardness test (HRC) was performed with the help of a TINIUS Olsen digital machine at 150 kg load.

Nano-indentation tests were executed to determine the elastic properties of the samples. All the tests were performed with the help of a Hysitron TI 750 nano-indentation machine equipped with Berkovich indenter. Moreover, the tip radius of the Berkovich indenter was 150 nm. The peak load of 8000 μN was applied during loading. However, the loading rate was kept at1600 μN/s. Additionally, the withhold time between loading and unloading was kept at 2 s for the measurement of nano-hardness. In the nano-indentation test, depth recovery ratio (η_h_) is used to determine the elastic behavior of material [26]. The mathematical equation for the depth recovery ratio (η_h_) is shown in Eq. (5) [26].

(5) $$\eta_{h}=\frac{h_{\max}-h_{r}}{h_{\max}}$$

where h_max_ is the maximum penetration depth, and h_r_ is the residual depth after unloading.

The microstructure of the samples was observed by a field emission scanning electron microscopy (Fe-SEM), Nova Nano SEM 450. The etching of the polished high carbon steels samples was done using a 3% Nital (3% nitric acid and 97% ethanol) solution. The statistical analysis of the microstructure of the pearlitic samples was done with the help of ImageJ software. Bruker contour GT-K 3-D optical profilometer was used to measure the surface roughness parameters of the samples. The microstructures of the cavitated samples were also analyzed using field emission scanning electron microscopy (Fe-SEM). The morphology of the cavitated top surface was captured just after the cavitation test without any etching. However, the samples were bisected for the cross-sectional analysis. The cross-section of the bisected sample was polished and etched similar to the heat-treated samples before taking Fe-SEM micrographs. Optical metallography of the cross-section of the cavitated samples was also carried out using an optical microscope (Leica Microsystems DM6000 M).

### 2.3 Experimental setup for cavitation test

The cavitation erosion experiments were carried out as per the ASTM G32-16 standards. The major components of the ultrasonic vibratory device used to perform experiments were a generator, a converter, and a horn attached with a vibratory tip. There are two ways of performing these experiments, i.e., direct and indirect. The specimen was attached to the horn in the direct method. Contrarily, it was kept at a distance in the indirect method [27]. The quantification of data in the first method is easier than the second method because the former gives higher mass loss [28]. The sample in the first method is attached to the horn through the threaded assembly. The materials on which it is difficult to make a thread cannot be used in this method, and hence, this method is only limited to the ductile materials [2]. The indirect method is preferred over the direct method to compare the different classes of materials in the same operating conditions because no special arrangement like thread-making is required [2]. The indirect method was used in this work to perform all cavitation erosion tests. The process parameters of the tests are as follows: 700 W power, 20 kHz frequency, 50 μm peak to peak amplitude, and the distance between the tip of horn and the sample surface maintained between 1 and 2 mm. The freely aerated 3.5% NaCl solution was used to perform the tests, and the temperature was kept in the range of 25 ± 2 °C using ice-cubes. The ice-cubes were entirely enclosed in the plastic tubes so that only heat can be transferred without any exchange in mass. The auto-shutoff limit of 27 °C was set in the machine. The ice-cubes were exchanged with newer ice-cubes whenever the operating temperature reaches to 27 °C. The machine was again started after the solution temperature reaches to 23 °C.

**Fig. 2:**
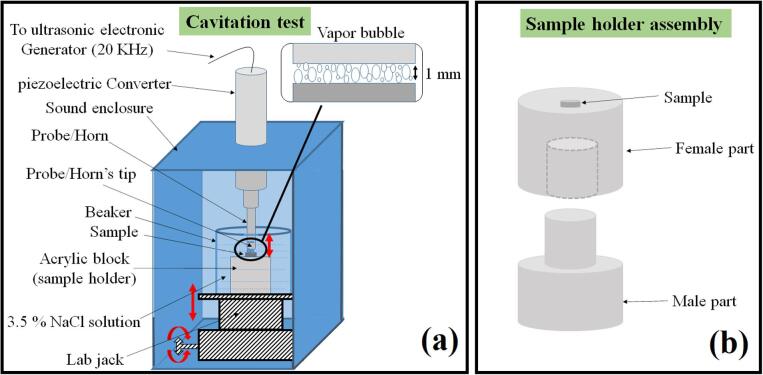
Schematic diagram of (a) the experimental setup (b) the sample holder assembly.

The schematic diagram of the experimental setup is shown in Fig. 2(a). The sample was fixed on the top of the sample holder. The sample holder consisted of two parts: a male and a female part. The male part was fixed to the beaker. However, the female part was attached to the male part using a push-fit assembly. The female part could be easily detached from the male part for the inspection of the sample. The sample holder assembly, along with the sample, is shown in Fig. 2(b).

The cavitation erosion tests of all the samples were carried out for a period of 10 h. The loss of material was measured after an interval of 1 h. However, the samples were dried before each mass measurement using a hot blower. If the samples were not dried correctly, they would show the loss in weight during measurement due to the evaporation of water vapor. The samples were dried in such a way that there should not be any variation in weight during weight measurement. Furthermore, the samples must show static weight for a period of 20 s during measurement.

The tests were repeated, and average data (Δm) was taken for further understanding. The total mass loss (M_i_) was calculated by adding the subsequent average mass loss of each interval. The mathematical expression of total mass loss (M_i_) is written in Eq. (6).

(6) $$M_{i}=\sum_{i=1}^{10}\Delta m_{i}$$

where: i = duration of test (hr); Δm_i_ = mass loss in i time period (mg).

The two basic parameters, which define the performance of material against the cavitation erosion, are mean depth erosion (MDE) and mean depth erosion rate (MDER) as per the ASTM-G32 standard [27]. The mathematical expressions of the MDE and the MDER for a particular period (i) are shown in Eqs. (3), (4), respectively.

(7) $${\mathrm{MDE}}_{i}=\sum_{i=1}^{10}\Delta {\mathrm{MDE}}_{i}=\frac{4\cdot M_{i}}{\rho \cdot \pi \cdot d_{p}^{2}}$$

(8) $$\mathrm{MDE}R_{i}=\frac{\Delta MDE_{i}}{\Delta t_{i}}.$$

where: ρ = density (mg/mm^3^); d_p_ = diameter of the sample (mm), Δt = test period.

## 3 Results and discussion

### 3.1 Microstructural characterization

**Fig. 3:**
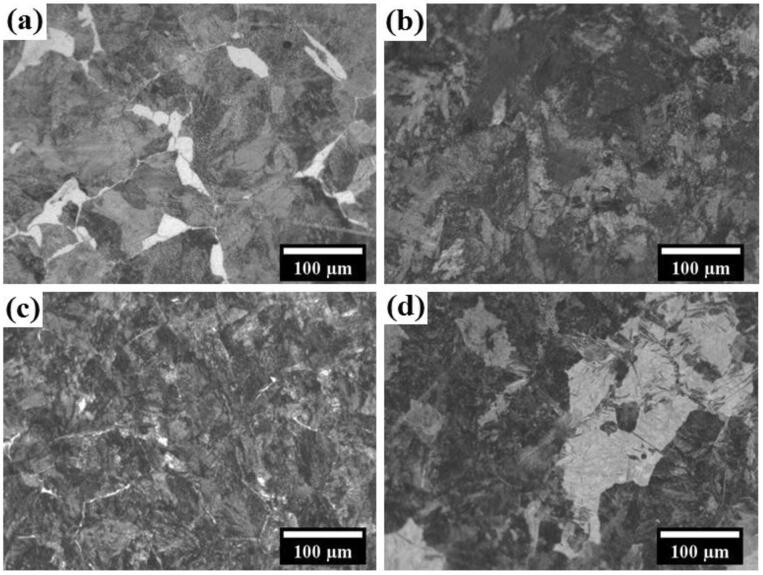
Optical micrographs of the pearlitic steels: (a) furnace-cooled, (b) as-received, (c) air-cooled, and (d) forced-air-cooled.

The optical micrographs of the furnace-cooled, as-received, air-cooled, and forced-air-cooled steels are shown in Fig. 3(a–d). The fineness of the microstructure increases with the rise in the rate of cooling. The furnace-cooled steel shows a very coarse microstructure, whereas the finest microstructure has been observed in the case of the forced-air-cooled steel. The grain growth and nucleation rate entirely depend on the rate of cooling. A high rate of cooling prohibits the grain growth and promotes the nucleation rate producing finer microstructure, and vice-versa for the slow rate of cooling. The pearlitic colony size also decreases as the rate of cooling increases. The pearlitic colony size has been measured using ImageJ software, and the average colony sizes of the pearlite have been found out to be 43.41 ± 7.3, 38.9 ± 8.9, 36.9 ± 9.3, 29.09 ± 2.8 μm for the furnace-cooled, as-received, air-cooled, and forced-air-cooled, respectively. The presence of sharp features within the bright regions in the case of the forced air-cooled steel is shown in Fig. 3(d). Furthermore, the presence of sharp features signifies the formation of some hard phases.

**Fig. 4:**
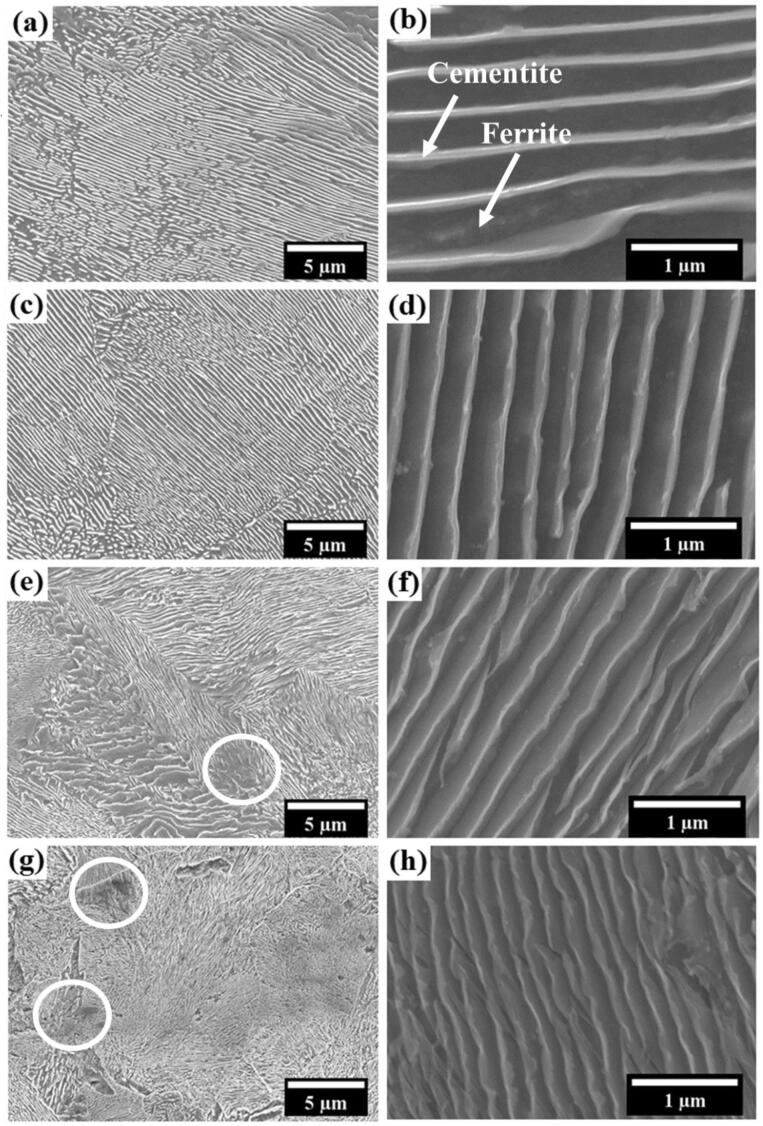
SEM micrographs of the different pearlitic steels at low and high magnifications: (a and b) furnace-cooled, (c and d) as-received, (e and f) air-cooled, and (g and h) forced-air-cooled.

The microstructures of all the samples have also been observed at higher magnification. The SEM micrographs of all the pearlitic steels are shown in Fig. 4(a–h). The cementite and ferrite phases are shown in Fig. 4(b) by arrowheads. The average cementite lamella thickness, ferritic lamella thickness, and the interlamellar spacing between the cementite lamella are shown in Table 1. Furthermore, from the statistical analysis, it has been observed that the average cementite lamella thickness, ferritic phase thickness, and the interlamellar spacing between the cementite lamella reduce in the following order: furnace-cooled, as-received, air-cooled and forced-air-cooled. However, the hardness of the pearlitic steels increases as the interlamellar spacing decreases. Further, it is clear from Table 1 that the interlamellar spacing decreases as the rate of cooling increases. Because of that, the highest and lowest hardness values have been found out in the case of the forced-air-cooled and the furnace-cooled pearlitic steel, respectively. As it is already known that both are pearlitic steels having the same composition, still they show vast difference in their respective hardness values due to the fineness of their microstructures. Furthermore, the manufacturing of the as-received rail involves the hot rolling followed by air cooling. Based on the microstructural and mechanical analysis, as shown in Table 1, it has been observed that the properties of the as-received rail steel lie in between the furnace-cooled and the air-cooled steel. Moreover, the increase in the hardness of different pearlitic steel is also attributed to ferrite to the cementite area ratio. Ferrite to cementite area ratio decreases as the rate of cooling increases in the following order: furnace-cooled, as-received, air-cooled, and forced-air-cooled. Furthermore, the proportion of the harder cementite phase also increases as the ferrite to cementite area ratio decreases. Finally, the increase in the fraction of the harder phase ultimately increases the hardness of steel.

**Table 1:** Statistical analysis of the microstructures of the pearlitic steels.

Process	Phase of steel	Average colony size (μm)	Cementite lamella thickness (nm)	Interlamellar spacing (nm)	Ferrite lamella thickness (nm)	Ferrite/cementite area ratio	Hardness (HRC)
Furnace-cooled	Pearlite (Coarse)	43.4 ± 7.3	71.5 ± 7.4	316.3 ± 31.1	244.8 ± 30.2	3.4	26.4 ± 0.6
As-received	Pearlite (medium)	38.9 ± 8.9	65.0 ± 6.8	279.0 ± 24.0	214.0 ± 23.9	3.3	30.1 ± 0.3
Air-cooled	Pearlite (fine)	36.9 ± 9.3	55.9 ± 6.1	203.4 ± 24.5	147.5 ± 26.4	2.6	41.1 ± 0.2
Forced-air-cooled	Pearlite (very fine)	29.0 ± 2.8	44.0 ± 4.3	155.9 ± 9.8	111.9 ± 11.2	2.5	63.3 ± 0.6

The signature of quasi-pearlite is also present during the higher cooling rate of the near eutectoid steel [29]. The signature of quasi-pearlite has not been observed in the case of the furnace-cooled steel. The probability of the formation of quasi pearlite increases in the following order: as-received, air-cooled, and forced-air-cooled [21]. The presence of quasi-pearlite also endorses the enhanced hardness of the air-cooled as well as the forced-air-cooled steel. The sign of quasi-pearlite is prominently evident in the air-cooled and the forced-air-cooled steels, and shown in Fig. 4(e, g) by circles.

### 3.2 Nano-indentation test

**Fig. 5:**
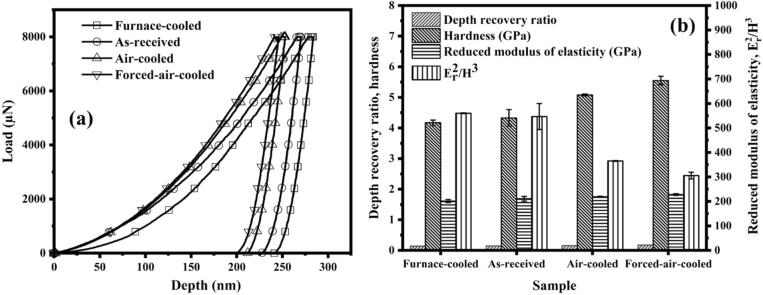
Plots obtained from the nano-indentation for the different steels: (a) load vs. displacement plots, and (b) $E_{r}^{2}/H^{3}$ ratio of different pearlitic steels.

The load versus displacement plots of the heat-treated steels along with the as-received steel are shown in Fig. 5(a), and the nano-indentation parameters calculated from the load vs. displacement plots are shown in Table 2. The indentation depth represents the deformation of different pearlitic steels under the same load. The maximum indentation depth represents the total deformation at the end of loading, whereas residual indentation depth represents the total deformation after the removal of load [30]. The highest and lowest values of maximum and residual indentation depth have been obtained in the case of the furnace-cooled and the forced-air-cooled steel, respectively. The indentation depths of the as-received and the air-cooled steel lie between the furnace-cooled and the forced-air-cooled steel. Furthermore, the maximum and residual indentation depths decrease similar to the interlamellar spacing in the following order: furnace-cooled, as-received, air-cooled, and forced-air-cooled. Whereas, the variation of nano hardness, as shown in Fig. 5(b) shows increasing trend in the following order: furnace-cooled, as-received, air-cooled, and forced-air-cooled. Hence, finer and harder pearlitic steel has a greater ability to resist the deformation as compared to the coarser pearlitic steel.

**Table 2:** Indentation parameters derived from the nano-indentation test for the pearlitic steels.

Sample	H_maximum_ (nm)	H_residual_ (nm)	Depth recovery ratio (η_h_)	Hardness (H) (GPa)	Reduced modulus of elasticity (E_r_) (GPa)	E_r_^2^/H^3^
Furnace-cooled	281.23 ± 2.82	242.36 ± 1.80	0.1382 ± 0.0022	4.17 ± 0.09	201.58 ± 6.24	560.24 ± 1.58
As-received	275.68 ± 9.39	235.2055 ± 7.85	0.1468 ± 0.0005	4.33 ± 0.27	210.98 ± 9.56	552.95 ± 53.26
Air- cooled	253.75 ± 0.80	214.2198 ± 0.84	0.1558 ± 0.0006	5.08 ± 0.03	218.85 ± 2.4	365.32 ± 1.54
Forced-air- cooled	243.55 ± 3.54	201.7332 ± 4.73	0.1718 ± 0.0073	5.55 ± 0.14	228.35 ± 3.5	305.54 ± 13.75

The values of the depth recovery ratio are calculated to determine the elastic characteristics of the material, and the higher value of the depth recovery ratio indicates the better elastic properties [26]. The values of depth recovery ratio have increased in the following order: furnace-cooled, as-received, air-cooled, and forced-air-cooled. The variation of depth recovery ratio is also shown in Fig. 5(b). The forced-air-cooled pearlitic steel has the highest value of depth recovery ratio. Hence, it has the maximum ability to deform elastically without going to the permanent deformation zone. The reduced modulus of elasticity for each steel is shown in Table 2. It represents the total deformation of the indenter tip and the sample material [30], and it increases in the following order: furnace-cooled, as-received, air-cooled, and forced-air-cooled. The variation of reduced modulus of elasticity for different steels is shown in Fig. 5(b). Hence, the forced-air-cooled steel has the best elastic properties in terms of depth recovery ratio and reduced modulus of elasticity.

The ratio of $E_{r}^{2}/H^{3}$ has been calculated to determine the wear resistance of the pearlitic steels [31], where E_r_ and H represent the reduced modulus of elasticity and hardness of the steel, respectively. The variation of the $E_{r}^{2}/H^{3}$ ratio is shown in Fig. 5(b). It is well known that the hardness of the material is generally related to the wear performance of the material [13]. However, the ratio of modulus to hardness is a better indicator than hardness itself [31]. The reduction in the values of $E_{r}^{2}/H^{3}$shows the enhancement in the wear resistance of the material [31]. The values of the $E_{r}^{2}/H^{3}$ have decreased in the following order: furnace-cooled, as-received, air-cooled, and forced-air-cooled. The forced-air-cooled steel exhibits the minimum value of $E_{r}^{2}/H^{3}$, thus leading to the maximum resistance against the cavitation erosion wear. It has been concluded after the nano-indentation test that the forced-air-cooled steel holds excellent mechanical properties such as depth recovery ratio, the ratio of $E_{r}^{2}/H^{3}$, reduced modulus of elasticity, and hardness in comparison to the rest of the pearlitic steels.

The Rockwell hardness, as shown in Table 1, can be directly correlated with the nano hardness listed in Table 2. It means that the values of macro and micro-hardness increase with the fineness of pearlite. The depth recovery ratio also increases along with the increase in the macro and micro-hardness. Hence, it can be concluded that the finer pearlitic has a greater ability to deform under external load without going into permanent deformation. Moreover, it also has greater hardness. Both properties are very beneficial for a material employed in a cavitation-prone environment. The reduced modulus of elasticity increases as the fineness of pearlitic steel increases from furnace-cooled to forced-air-cooled in the following order: furnace-cooled, as-received, air-cooled, and forced-air-cooled. Furthermore, the ratio $E_{r}^{2}/H^{3}$ decreases as the interlamellar decreases. The decrease in the ratio of $E_{r}^{2}/H^{3}$ indicates the improvement in the erosive wear performance of the steel. Hence, it can be concluded that the properties like hardness, depth recovery ratio, $E_{r}^{2}/H^{3}$ ratio, etc., have been improved by only changing the microstructure of the pearlitic steel from coarse to fine.

### 3.3 Cavitation erosion tests

**Fig. 6:**
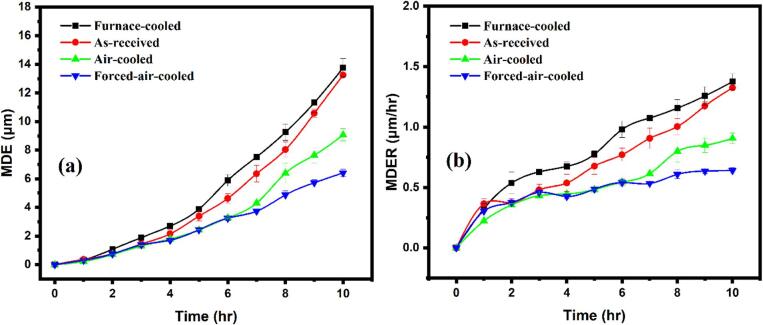
Variation of (a) the MDE and (b) the MDER with respect to time for different pearlitic steels.

The variations of the mean depth of erosion (MDE) and the mean depth erosion rate (MDER) corresponding to erosion time are shown in Fig. 6(a and b), respectively. The MDE represents the growth of pit depth related to erosion time, whereas the MDER represents the nature of the cavitation process, which is related to the severity of the cavitation damage. In other words, the MDER represents the slope of the MDE versus the erosion time plot. Higher is the MDER, lower would be the resistance to erosion or cavitation damage. The incubation periods shown by the furnace-cooled, as-received, air-cooled, and forced-air-cooled are 1 h, 2 h, 4 h, and 4 h, respectively. The incubation period is identified as the time duration from the starting of the test to the start of the accelerating stage [27]. During the incubation period, the samples show negligible erosion as compared to the later stages. In this work, the incubation period has been taken as 0.3 µm/h. The rapid growth in the MDER has been observed for the furnace-cooled and the as-received pearlitic steels after the incubation period. However, a slow rise in the MDER has been noticed in the case of the air-cooled and the forced-air-cooled steel after the incubation period. The values of the MDER after 10 h for the furnace-cooled, the as-received, the air-cooled, and the forced-air-cooled steels are 1.37 ± 0.06, 1.32 ± 0.01, 0.90 ± 0.04, and 0.64 ± 0.02 µm/h, respectively. Moreover, the cavitation erosion resistance of the material is defined as reciprocal of MDER_max_ [13]. The values of the cavitation erosion resistance of the furnace-cooled, as-received, air-cooled, and forced-air-cooled have been calculated to be 0.72, 0.75, 1.10, 1.56 h/µm, respectively. Thus, the air-cooled and the forced-air-cooled show better resistance against the cavitation erosion as compared to the furnace-cooled and the as-received pearlitic steels. The latter (the as-received and the furnace-cooled) have similar MDER values suggesting that their erosion resistance is similar but poorer than the other two. However, if the variation of the MDER for the steels is closely observed (Fig. 6(b)), the furnace-cooled steel shows higher MDER throughout the time scale, followed by the as-received, the air-cooled, and finally the forced air-cooled steel.

The furnace-cooled steel shows the maximum value of the MDE, and it means that the depth of the pits is maximum in the case of the furnace-cooled steel with coarse pearlite. In comparison, the minimum depth of pits has been noticed in the case of the forced-air-cooed steel, consisting of finest pearlite and the presence of quasi-pearlite. The MDE values observed for the furnace-cooled, the as-received, the air-cooled, and the forced-air-cooled are 13.75 ± 0.64, 13.24 ± 0.10, 9.06 ± 0.42, and 6.41 ± 0.24 µm, respectively, at the end of the test. The as-received steel shows cavitation erosion performance close to the furnace cooled steel, whereas the air-cooled steel shows its cavitation erosion performance poorer to the forced-air-cooled steel. However, Fig. 6(a) shows the trace of the data for different time range, and it is very clear that the variation of MDE is similar to the variation of MDER for all the steels. The furnace-cooled steel shows the maximum MDE, and the forced-air-cooled steel shows the minimum MDE. The as-received steel has a higher MDE than the air-cooled one.

The variations in the MDE and the MDER for the steels are closely related to the microstructural features (Table 1) as well as the depth recovery ratio and the $E_{r}^{2}/H^{3}$ ratio (Table 2). The average colony size, cementite lamellar thickness, and interlamellar spacing for the four types of steels decrease in the following sequence: furnace-cooled, as-received, air-cooled, and forced-air-cooled (Table 1). The hardness of the steels thus varies in the opposite sequence. Interestingly, the cavitation resistance of the four types of steels also varies in the increasing sequence as furnace-cooled – as-received – air-cooled – forced-air-cooled. Therefore, the cavitation resistance is directly related to the hardness of the steels, and the finer the microstructural features, the better is the cavitation resistance of pearlitic steels. The cavitation erosion resistance also increases as the ferrite to cementite ratio decreases. The decrease in ferrite to cementite ratio leads to an increase in the harder cementite phase. The cementite phase has a greater ability to combat the powerful pressure waves as compared to the ferritic phase, since cementite is a harder phase. In general, it has been observed that in the matrix of hard and soft phase, the softer phase is more prone to cavitation erosion damage as compared to the harder phase [32].

On the other hand, the depth recovery ratio of the steels is inversely related to the $E_{r}^{2}/H^{3}$ ratio (Table 2). Consequentially, the variation of cavitation resistance of the steels is inversely related to the $E_{r}^{2}/H^{3}$ ratio and directly related to the depth recovery ratio. This is quite understandable. The higher the depth recovery ratio, the higher would be the elastic recovery of the deformed region due to cavitation leading to the shallow depth or lower MDE. Similarly, lower the value of the $E_{r}^{2}/H^{3}$ ratio, higher would be the resistance offered by the substrate material to the cavitation influenced indentation.

### 3.4 Morphology of the surface after cavitation test

**Fig. 7:**
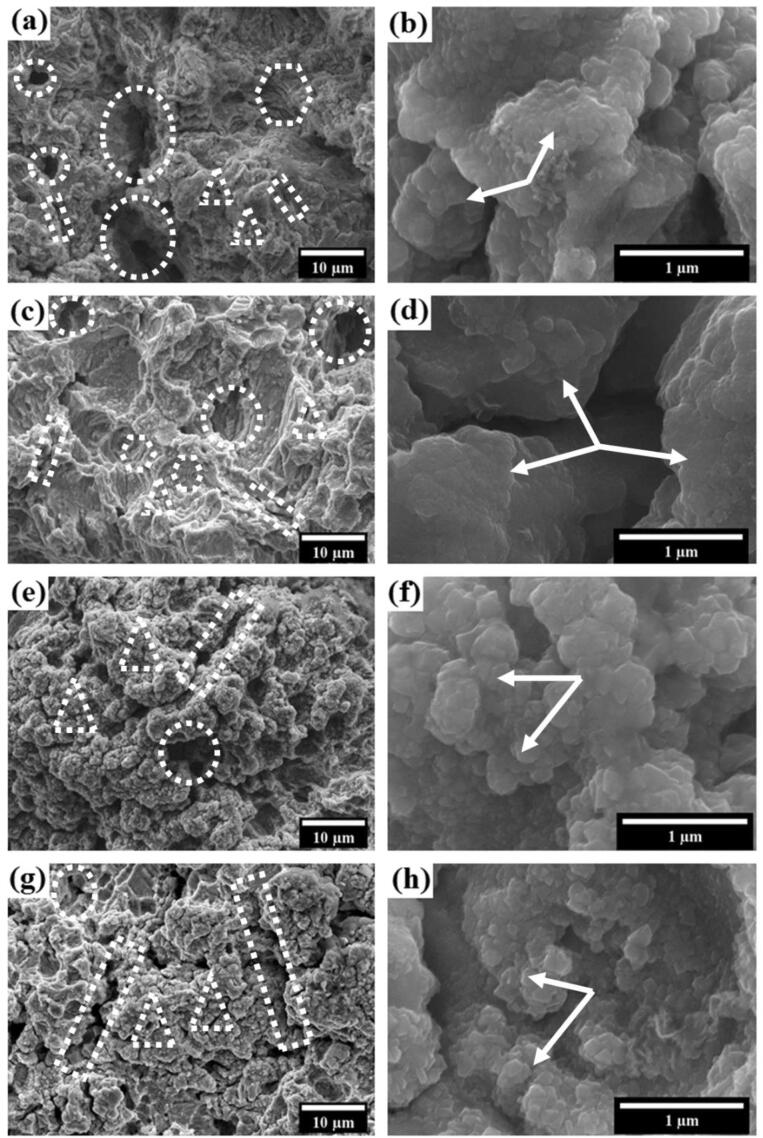
SEM micrographs of the surfaces of different pearlitic steels after cavitation test at low and high magnification: (a and b) furnace-cooled, (c and d) as-received, (e and f) air-cooled, and (g and h) forced-air-cooled.

The SEM micrographs of the cavitated surfaces of the heat-treated steel samples, along with the as-received steel sample, are shown in Fig. 7(a–h). Micro-cracks, pits, craters, and striations are visible in all the samples. The micro-cracks, pits, craters, and striations are shown in Fig. 7(a–h) by rectangles, triangles, circles, and hexagons, respectively. The average size of the craters and crack length is shown in Table 3. The large size craters are visible at the interface of pearlitic colonies in the case of the furnace-cooled steel as shown in Fig. 7(a). The damaged surface of the furnace-cooled steel also consists of pits, cracks, and striations. Moreover, the surface is predominantly dominated by large and small size craters. These craters are mainly responsible for the high MDE and the MDER of the furnace-cooled steel. In the case of the as-received steel, the fractured surface is shown in Fig. 7(c). The fractured surface mostly contains the medium size craters along with cracks and pits. However, the size of the craters is comparatively smaller than those of the furnace-cooled steel. The cavitated surface of the air-cooled and the forced-air-cooled steel are shown in Fig. 7(e) and (g), respectively. The surface of both the steels is primarily dominated by the smaller size pits and cracks. The large size craters are not visible in Fig. 7(e, g).

**Table 3:** Statistical analysis of crater size and crack length of different pearlitic steels.

Sample	Crack length (µm)	Crater size (µm)
Furnace-cooled	5.16 ± 0.08	13.51 ± 1.19
As-received	7.95 ± 0.02	9.19 ± 0.24
Air-cooled	13.84 ± 2.86	6.04 ± 0.99
Forced-air-cooled	25.52 ± 2.98	4.64 ± 0.80

The microstructures of the damaged surfaces of different pearlitic steels very well correlate with the cavitation erosion resistance of the respective steels. The finer pearlitic steels have greater cavitation erosion resistance due to the presence of small pearlitic colonies. Moreover, the sizes of the craters and cracks also decrease from the furnace-cooled steel to the forced-air-cooled steel in the following sequence: furnace cooled – as-received – air-cooled – forced-air-cooled. The higher magnification Fe-SEM images of the damaged surface of the furnace-cooled and the as-received are shown in Fig. 7(b) and (d), respectively. It has been observed from Fig. 7(b and d) that material has been removed without fragmenting into smaller pieces due to larger pearlitic colony size. The large damaged pearlitic colonies are shown in Fig. 7(b) and (d) by arrowheads. However, in the case of the air-cooled and the forced-air-cooled steels, smaller size pearlitic colonies are shown by arrowheads in Fig. 7(f) and (h), respectively. Breaking of the large pearlitic colony in case of the furnace-cooled, and the as-received steels lead to their higher cavitation damage. On the contrary, finer pearlite colony size helps in providing better cavitation resistance to the air-cooled and the forced-air-cooled steels.

**Fig. 8:**
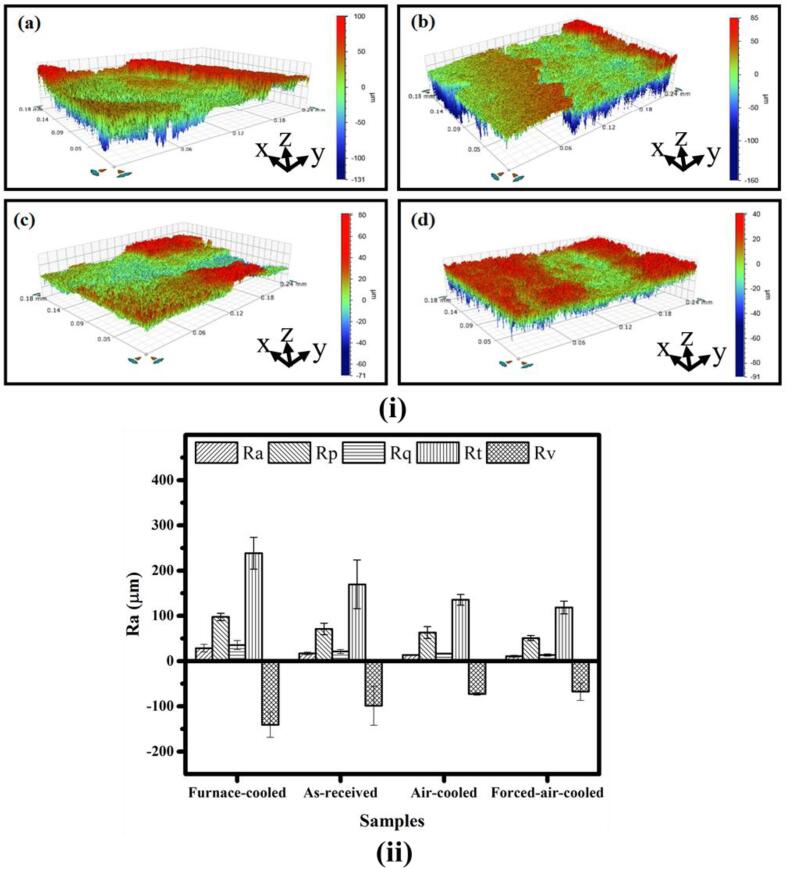
(i) 3D plots of the damaged surfaces after cavitation test of the pearlitic steels: (a) furnace-cooled, (b) as-received, (c) air-cooled, and (d) forced-air-cooled. (ii) Surface roughness parameters of the damaged surfaces after the cavitation erosion test of the pearlitic steels.

The surface profiles of the damaged surfaces, as captured by the optical profilometer, are shown in Fig. 8(i). Moreover, the surface roughness parameters, such as average surface roughness (R_a_), root mean square surface roughness (R_q_), maximum peak height (R_p_), Maximum valley height (R_v_), and maximum peak to valley height (R_t_) extracted from the optical profilometer data, are shown in Fig. 8(ii). The maximum and minimum values of the average surface roughness have been noted for the furnace cooled and the forced air-cooled, respectively. The surface roughness parameters of the cavitated surface are also presented in Table 4. The deviation in the surface roughness parameters is more in the case of the furnace-cooled and the as-received steels as compared to the air-cooled and the forced-air-cooled steels. Because of this, the error bars shown in Fig. 8(ii) are much larger for the furnace-cooled and the as-received steel as compared to the air-cooled and the forced-air-cooled steels. Moreover, the absolute values of all the surface roughness parameters, as indicated in Table 4, decrease in the following order: furnace cooled – as-received – air-cooled – forced-air-cooled steels. It has also been observed from the surface roughness values in Fig. 8(ii) that uniform cavities are noticeable in the case of the fine (air-cooled) and very fine pearlitic steels (forced-air-cooled), whereas non-uniformed cavities are prominent in the case of coarse (furnace-cooled) and moderately fine pearlitic steel (as-received). In the case of the furnace-cooled and the as-received steel, the softer ferrite phase is more exposed to powerful waves due to large interlamellar spacings (Table 1). This results in a higher mass loss from the ferrite phase as compared to the harder cementite phase. Whereas, in the case of the air-cooled and the forced-air-cooled steel, the exposer of the ferrite phase decreases.

**Table 4:** Surface roughness parameters derived from optical profilometer for the pearlitic steels.

Sample	Surface roughness parameters				
	Average roughness, R_a_ (μm)	RMS roughness, R_q_ (μm)	Maximum profile peak height, R_p_ (μm)	Maximum profile valley depth, R_v_ (μm)	Maximum height of the profile, R_t_ (μm)
Furnace-cooled	28.48 ± 8.59	35.33 ± 10.05	97.71 ± 8.11	−140.79 ± 27.88	238.50 ± 35.16
As-received	16.85 ± 3.16	21.26 ± 4.64	71.03 ± 12.74	−98.63 ± 43.18	169.67 ± 53.74
Air-cooled	13.25 ± 1.02	16.46 ± 1.34	63.20 ± 13.14	−72.52 ± 2.81	135.72 ± 11.87
Forced-air- cooled	10.82 ± 2.11	13.55 ± 2.62	50.92 ± 5.42	−67.44 ± 19.47	118.36 ± 14.06

**Fig. 9:**
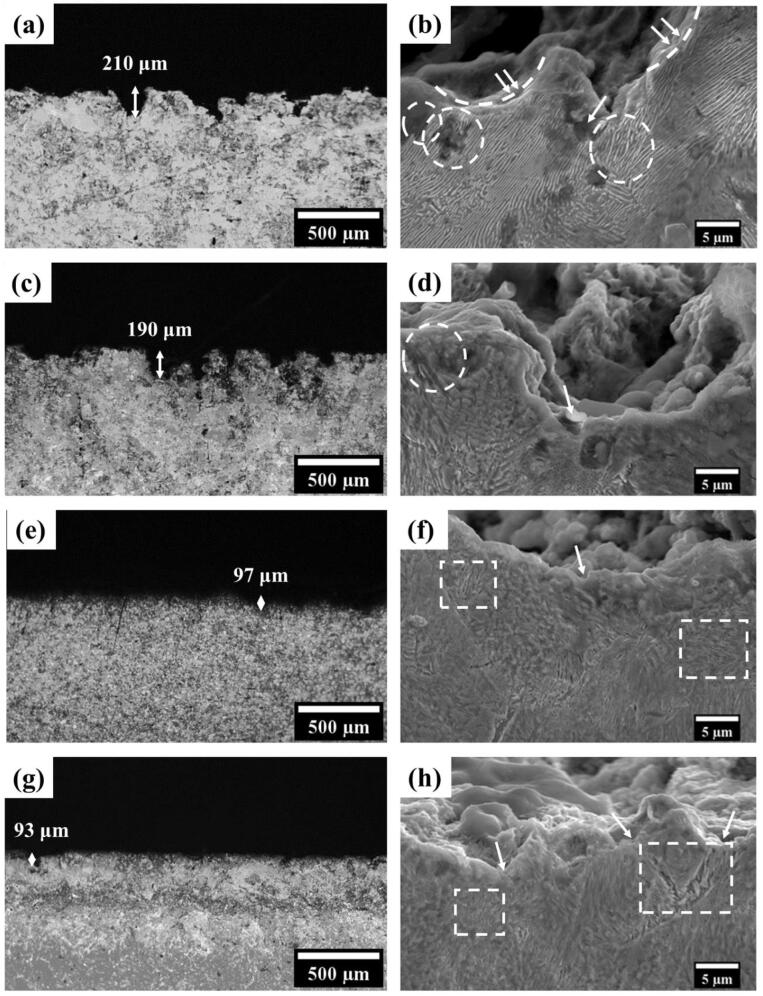
Low (optical) and high (SEM) magnification micrographs of the cross-sections of the pearlitic steels: (a and b) furnace-cooled, (c and d) as-received, (e and f) air-cooled, and (g and h) forced-air-cooled.

Fig. 9(a, c, e, and g) show the optical cross-sectional images of the eroded portions for the furnace-cooled, as-received, air-cooled, and forced-air-cooled steels after the end of the 10 h of cavitation experiment indicating the maximum depths of attack of 210, 190, 97 and 93 µm, respectively. This directly advocates that the increasing cavitation resistance of the steels is directly reflected by the decreasing maximum depth of attack in the following sequence: furnace cooled – as-received – air-cooled – forced-air-cooled. Fig. 9(b, d, f, and h) show the SEM cross-sectional microstructures along the cavitation damaged portions for the four kinds of steels after 10 h of cavitation test. The attack patterns can be correlated to the relative cavitation resistance of the steels. The damage follows along with the interfaces between two adjoining pearlite colonies, as indicated by arrowheads in Fig. 9(b, d, f, and h). This also corroborates well with the surface morphology of the damaged portions in Fig. 7(b, d, f, and h). Moreover, the alignment of the pearlite lamellar structure has a specific effect on the damage of the steels. If the progress of the burst wave is parallel to the cementite alignment, the damage cannot progress easily. This is quite well understood from one such example shown in Fig. 9(b), where the progress of burst wave is shown as a broken half-circle, and the side portion as indicated by double arrowhead is parallel to the wave movement. The broken half-circle is not progressing deep into the material since it provides resistance by the reinforcing effect of the parallel alignment of the hard cementite lamellae. The influence of galvanic dissolution of anodic ferrite lamellae in electrochemically connected with cathodic cementite lamellae is also very much reflected (shown as dotted circles in Fig. 9(b and d). It is interesting to mention that the specific dissolution of the ferrite lamellae in the steels with finer microstructures (air-cooled and forced-air-cooled) is not that prominent as it is in the case of the furnace-cooled and the as-received steels, where the interlamellar spacing is large and the thickness of the ferrite lamellae is also large (Table 1). This leads to protruded cementite lamellae because of the dissolution of side-by ferrite lamellae, and these protruded cementite plates later break down due to burst waves during cavitation, allowing the progress of the damage. This matches well with the observation by Katiyar et al. [21] during dynamic polarization of differently cooled steels of the same composition, where the dissolution of ferrite is greater in the case of coarse pearlite than finer pearlite. This also leads to higher dissolution and thus cavitation damage in the case of the furnace-cooled and the as-received steels as indicated by the depth of attack shown in Fig. 9(a, c, e, and g) than the cases with finer microstructures. Finally, the presence of quasi-pearlite in the air-cooled and the forced-air-cooled steels could impart added resistance to the cavitation damage for these two types of steels. This is quite evident in Fig. 9(f and h). Wherever the progress of bursting wave interacts with the regions with quasi-pearlite, which is a hard phase, the progress is hindered. This is shown by the dotted rectangle in Fig. 9(f and h). Hence, it is very clear that the cavitation damage of the four types of pearlitic steels is strongly related to the microstructural features, their $E_{r}^{2}/H^{3}$ ratio and the depth recovery ratio.

## 4 Conclusions

The air-cooled and the forced-air-cooled steel show better cavitation erosion resistance as compared to the furnace-cooled and the as-received steel primarily due to higher hardness and fineness of the pearlitic phase in the former steels. It has been noticed that as the rate of cooling increases, the interlamellar spacing decreases. The decrease in interlamellar spacing results in the finer microstructure of the pearlitic steel. Subsequently, the mechanical properties, such as hardness, depth recovery ratio, and the $E_{r}^{2}/H^{3}$ ratio, have also improved. Moreover, the smaller size of pearlitic colonies further enhances the cavitation erosion resistance of the air-cooled and the forced-air-cooled steel as compared to the furnace-cooled and the as-received steel. Increase in the depth recovery ratio and decrease in the $E_{r}^{2}/H^{3}$ ratio also increase cavitation erosion resistance. The presence of quasi-pearlite also imparts an excellent resistance against the growth of the crack.

## Declaration of Competing Interest

The authors declare that they have no known competing financial interests or personal relationships that could have appeared to influence the work reported in this paper.
